# Avoidable factors associated with maternal death from postpartum haemorrhage: a national Malawian surveillance study

**DOI:** 10.1136/bmjgh-2024-015781

**Published:** 2025-01-09

**Authors:** Jennifer Riches, James Jafali, Hussein H Twabi, Yamikani Chimwaza, Marthe Onrust, Rosemary Bilesi, Luis Gadama, Fannie Kachale, Annie Kuyere, Lumbani Makhaza, Regina Makuluni, Laura Munthali, Owen Musopole, Chifundo Ndamala, Deborah A Phiri, Arri Coomarasamy, Abi Merriel, Catriona Waitt, Maria Lisa Odland, David Lissauer

**Affiliations:** 1 Women's and Children's Health, University of Liverpool, Liverpool, UK; 2 Maternal and Fetal Health Group, Malawi-Liverpool-Wellcome Trust Clinical Research Programme, Blantyre, Malawi; 3 Reproductive Health Directorate, Government of Malawi Ministry of Health, Lilongwe, Malawi; 4 Kamuzu University of Health Sciences, Blantyre, Malawi; 5 Malawi-Liverpool-Wellcome Trust Clinical Research Programme, Blantyre, Malawi; 6 Institute of Metabolism and Systems Research, University of Birmingham, Birmingham, UK; 7 Infectious Diseases Institute, Makerere University, Kampala, Uganda; 8 Department of Public Health and Nursing, Norwegian University of Science and Technology, Trondheim, Norway

**Keywords:** Maternal health, Global Health, Epidemiology, Health systems, Obstetrics

## Abstract

**Background:**

Despite strong evidence-based strategies for prevention and management, global efforts to reduce deaths from postpartum haemorrhage (PPH) have failed, and it remains the leading cause of maternal mortality. We conducted a detailed review of all maternal deaths from 33 facilities in Malawi to identify health system weaknesses leading to deaths from PPH.

**Methods:**

Data were collected regarding every maternal death occurring across all district and central hospitals in Malawi. Deaths occurring from August 2020 to December 2022 were reviewed by multidisciplinary facility-based teams who compiled case narratives from clinical notes and then subsequently reviewed by obstetricians to confirm the cause of death according to international criteria. Data were summarised using proportions/frequencies, comparisons made using χ^2^ or Wilcoxon rank sum tests, and logistic regression conducted to calculate ORs with CIs.

**Results:**

PPH was the cause of 20.4% of maternal deaths. Most deaths from PPH occurred within 24 hours of birth (80.0%), among women who had been referred to a higher-level facility (57.0%) and were admitted in stable condition (60.0%). Vacuum births carried an increased risk of death from PPH (OR 4.25 (95% CI 1.15 to 20.13, p=0.039)). Detailed reviews identified that deaths from PPH were more likely to be associated with factors such as ‘lack of obstetric lifesaving skills’ (26.7% vs 10.1%, p<0.001), ‘inadequate monitoring’ (51.5% vs 40.7%, p=0.012) and ‘communication problems between facilities’ (11.5% vs 6.2%, p=0.019) than deaths from other causes.

**Conclusions:**

Our analysis represents the largest published review of maternal deaths from PPH. We demonstrate that key health system weaknesses are contributing to these preventable maternal deaths. Case reviews conducted by multidisciplinary facility-based teams identified common and recurrent avoidable factors associated with deaths from PPH. Global efforts must now be focused on strategies that address these weaknesses, strengthening health systems and empowering healthcare workers to reduce maternal deaths from PPH.

**WHAT IS ALREADY KNOWN ON THIS TOPIC**

- Postpartum haemorrhage (PPH) is a leading cause of maternal death worldwide, disproportionately affecting low-resource African settings.
- To date, evidence synthesis has focused on determining obstetric risk factors for PPH and developing effective medical and surgical strategies for prevention and management. Few published studies have set out to investigate the potentially avoidable health system factors that lead to maternal deaths from PPH in low-resource settings.

**WHAT THIS STUDY ADDS**

- We found that most women who died of PPH in this setting died within healthcare facilities and that avoidable health system factors were more frequently and significantly associated with deaths from PPH than with deaths from other causes.

**HOW THIS STUDY MIGHT AFFECT RESEARCH, PRACTICE OR POLICY**

- Findings from our study can be used to understand the gap that exists between the dissemination of evidence-based strategies to prevent and manage PPH, and the effective implementation of such strategies in low-resource real-world settings.

## Introduction

 Postpartum haemorrhage (PPH) is the leading cause of maternal mortality worldwide, causing a quarter of all maternal deaths^1^ despite the existence of evidence-based medical and surgical strategies for its prevention and management.^2^,^6^ PPH may occur after any birth, but the distribution of maternal deaths from this cause demonstrates significant global inequity, with the majority of deaths from PPH occurring in the WHO African region.^7^ The WHO Postpartum Haemorrhage Summit 2023 outlined a road map and call to action to combat PPH.^8^ The summit asked the questions, ‘where is implementation breaking down?’ and ‘what is needed to get evidence-based interventions to women who need them most?’ Priority research topics were agreed to generate further evidence on the optimum implementation of strategies to prevent and manage PPH, including the need for better understanding of barriers and facilitators to effective implementation, and capacity building for front-line providers.

With these questions in mind, we conducted a secondary analysis of routinely collected maternal mortality data from a digital maternal health surveillance platform in Malawi, a low-income country in the African Region with a maternal mortality ratio of 381 per 100 000 live births.^9^ We aimed to determine the burden of deaths from PPH, characterise women dying of PPH and analyse avoidable factors involved in deaths from this cause to better understand existing barriers and facilitators to effective implementation of PPH prevention and management strategies in resource-constrained settings.

## Methods

### Study setting

Data for this study were collected from 33 healthcare facilities across Malawi, including all 4 central (tertiary-level) hospitals and all 27 district (secondary-level) hospitals (online supplemental figure S1). Deaths occurring outside of these facilities are reported by the corresponding district hospital, and therefore, also included in our analysis.

We conducted a retrospective analysis of individual-level maternal mortality data routinely collected using a digital surveillance platform. The observation period was between 1 August 2020 and 31 December 2022.

Participants eligible for enrolment in the study included all women who died from a cause related to or aggravated by pregnancy or its management or within 42 days of the end of the pregnancy, in keeping with the WHO definition of maternal death.^10^ Maternal deaths were included if they had been audited by a local maternal death surveillance and response (MDSR) committee to provide information regarding the cause and circumstances of the death. Deaths which were reported but not audited were excluded due to lack of sufficient detail. We aimed to include all maternal deaths occurring in the observation period in our sample.

### Data collection

Data were collected using a digital maternal health surveillance platform (MATSurvey), established by the Malawi-Liverpool-Wellcome Research Programme and the Ministry of Health (MOH) of Malawi in 2020 to digitalise and enhance the surveillance potential of routinely collected maternal health data.^11^ Maternal deaths occurring in healthcare facilities were identified by healthcare staff and reported to the MOH using a standard reporting tool. Information from the audit by the local MDSR committee was entered into data collection tablets (OpenDataKit V.1.21.0) and uploaded to the MATSurvey platform. This task was performed by ‘Safe Motherhood Coordinators’ at each study site, nurse-midwives trained by the MOH in case-finding and data collection. In the Malawian setting, most women deliver in hospital facilities. In the event of maternal death outside of the hospital, it is usual for the patient to be brought to the healthcare facility after death. All deaths must be registered, and where maternal deaths occurred outside of the government facility setting (including private facilities), they were reported to the facility or District Health Office and followed up by the Safe Motherhood Coordinator to gain maximum information from clinical notes detailing reports of what had happened prior to the patient’s death.

Case narratives describing each maternal death were compiled by MDSR committees. Minor details have been changed and cases merged to protect anonymity. Aggregate data from facilities, including information about the number of births, were used to calculate an average caesarean section (CS) rate and assisted vaginal birth rate. Data used in this analysis were fully anonymised and were available to the authors through permission from the MOH of Malawi and the College of Medicine Research Ethics Committee. Patient confidentiality and the ethical requirements of the committee were adhered to.

### Determining cause of death and avoidable factors

The cause of each maternal death was initially determined by local MDSR committees. The case narrative recorded for each death was independently reviewed by an obstetrician, mapping the cause of death to the WHO International Statistical Classification of Diseases categories,^10^ adapted for current use in Malawi by the MOH. The cause of death was defined using WHO principles as ‘the disease or condition that initiated the morbid chain of events leading to the death’.^10^ Where there were discrepancies, a second obstetric opinion was sought. Four categories (each with subcategories) were used to determine the avoidable factors associated with each death. These categories were predetermined by the MOH and linked to each case by local MDSR committees when reviewing deaths. They included ‘healthcare worker factors’ (related to healthcare worker practices such as monitoring, referral and timeliness of action), ‘administrative factors’ (related to resources, infrastructure, transport and communication), ‘patient/family factors’ (related to health-seeking behaviour, barriers to care) and ‘traditional birth attendant/community factors’. Each case could be ascribed to an unlimited number of avoidable factors.

### Data analysis

Categorical variables were summarised using frequencies and proportions, and continuous variables using medians and IQRs. Between-group analyses were conducted to compare women dying of PPH with women dying of other causes using χ^2^ tests for categorical variables and Wilcoxon rank sum test for non-normal continuous variables. We applied logistic regression analysis to estimate the odds (with 95% CIs) associated with dying of PPH compared with other causes of maternal death. Significance was determined at p value of less than 0.05. Analyses were performed by using R V.4.2.3 (21 March 2023).

Case narratives for maternal deaths were reviewed and excerpts were extracted and presented to highlight the most frequently occurring avoidable factors in deaths from PPH. An undirected, weighted network plot was produced to depict the relationship between avoidable factors.

### Patient

Given the nature of the data collection and analysis undertaken for this study, no patients were involved in the research process, nor was any public involvement solicited.

## Results

### Deaths from PPH

In total, 1162 maternal deaths were reported by facilities during the study period, and 809 audited deaths were included in the final analysis. PPH was the second-leading cause of maternal death (n=165 (20.4%) deaths), preceded by infection (n=201 (24.8%) of 809 deaths) and followed by eclampsia (n=108 (13.3%) deaths).

### Demographic and clinical characteristics of women who died of PPH

Table 1 shows the demographic and clinical characteristics of women who died of PPH compared with women who died of other causes.

**Table 1:** Demographic and clinical characteristics of women who died of postpartum haemorrhage (PPH) compared with women who died of alternative causes

Characteristic	PPH n=165 n (%)	Other n=644 n (%)	P value*
Age groups			0.3
<20 years	20 (12.1)	114 (17.7)	
20–25 years	46 (27.9)	187 (21.0)	
26–35 years	60 (36.4)	208 (32.3)	
>35 years	39 (23.6)	135 (21.0)	
Marital status			**0.016**
Married	136 (95.1)	500 (87.1)	
Single	5 (3.5)	60 (10.5)	
Other	2 (1.4)	14 (2.4)	
Education			**0.002**
None	30 (20.7)	195 (35.1)	
Primary	90 (62.1)	249 (44.9)	
Secondary	20 (13.8)	91 (16.4)	
Tertiary	5 (3.4)	20 (3.6)	
Parity			**0.007**
0	6 (4.9)	67 (14.6)	
1	30 (24.4)	111 (24.2)	
2	21 (17.1)	86 (18.7)	
3	18 (14.6)	78 (17.0)	
>3	48 (39.0)	117 (25.5)	
Timing of death			**<0.001**
Early pregnancy	0 (0)	52 (8.3)	
Antenatal	0 (0)	169 (27)	
Intrapartum	0 (0)	26 (4.1)	
Early postnatal (<24 hours)	132 (80.0)	152 (24)	
Late postnatal (24 hours to 42 days)	28 (17.0)	200 (32)	
Postnatal (unknown timing)	5 (3.0)	29 (4.6)	
Gestation at death			**<0.001**
<28 weeks	2 (1.3)	91 (16.0)	
28–31 weeks	8 (5.2)	66 (11.6)	
32–36 weeks	19 (12.3)	127 (22.4)	
37–42 weeks	125 (81.2)	284 (50.0)	
Mode of delivery			**0.004**
Caesarean section	68 (42.2)	208 (55.9)	
Vaginal birth	93 (57.8)	164 (44.1)	
ANC			**<0.001**
Did not receive ANC	6 (4.1)	107 (20.2)	
Received ANC	142 (95.9)	422 (79.8)	
HIV status			0.3
Negative	124 (90.5)	419 (87.5)	
Positive	13 (9.5)	60 (12.5)	
Location admitted from			0.5
Another facility	94 (57.0)	378 (60.1)	
Home/community	71 (43.0)	251 (39.9)	
Condition at admission			**<0.001**
Critically ill	51 (30.9)	390 (62.0)	
Dead on arrival	15 (9.1)	34 (5.4)	
Stable	99 (60.0)	205 (32.6)	

Bold type denotes statistical significance

* Pearson’s χ2 test; Fisher’s exact test.

ANC, antenatal care.

Most women who died of PPH were in the 26–35 years age group (n=60 (36.4%)) and were married (n=136 (95.1%)). The majority had either no education (n=30 (20.7%)) or were educated to only primary school level (n=90 (62.1%)). A minority of women were living with HIV (n=13 (9.5%)). Most women who died of PPH were multiparous (n=117 (95.1%)), with 39.0% (n=48) having given birth more than three times previously.

Just over half of all women who died of PPH were admitted to the facility where they died as a referral from another facility (n=94 (57.0%)); most arrived in stable condition (n=99 (60.0%)). Most had received antenatal care during their pregnancy (n=142 (86.1%)) and most women who died of PPH died between 37 and 42 weeks gestation (n=125 (81.2%)). The majority delivered vaginally (n=93 (57.8%)), although the proportion delivering by CS (n=68 (42.2%)) was higher than the overall proportion of women who delivered by CS across the included facilities (calculated as 16.0%). Most deaths from PPH occurred soon after delivery; 132 of 165 deaths (80.0%) occurred in the early postnatal period (<24 hours after birth) and 28 deaths (17.0%) in the late postnatal period (24 hours to 42 days following birth).

Compared with women who died of alternative causes, there was a significant difference in certain demographic characteristics of women who died of PPH including marital status, educational level and parity. There was no significant difference in age or HIV status. Among clinical characteristics, there were significant differences between the groups in the timing of death in relation to stage of pregnancy, gestation at death, mode of delivery, clinical condition on admission and whether antenatal care was received. ORs for death from PPH versus death from other causes for a range of demographic and clinical exposures are shown in table 2.

**Table 2:** ORs for death from PPH versus death from other causes for clinical exposures

	ORs (95% CIs)	P value
Age		
<20 years	–	–
20–25 years	1.40 (0.80; 2.53)	0.2
26–35 years	1.64 (0.96; 2.92)	0.079
>35 years	1.65 (0.92; 3.03)	0.10
Marital status		
Single	–	–
Married	**3.26 (1.41; 9.48)**	**0.013**
Other (divorced, widowed, separated)	1.71 (0.23; 8.91)	0.5
Education		
None	–	–
Primary	2.22 (1.41; 3.58)	<0.001
Secondary	1.40 (0.74; 2.60)	0.3
Tertiary	1.59 (0.50; 4.31)	0.4
Parity		
0	–	–
1	**2.65 (1.03; 8.22)**	**0.061**
2	2.46 (0.90; 7.90)	0.10
3	**3.03 (1.12; 9.68)**	**0.040**
>3	**4.62 (1.89; 13.9)**	**0.002**
Timing of death		
Early postnatal (<24 hours)	–	–
Late postnatal (>24 hours to 42 days)	**0.16 (0.10; 0.26)**	**<0**.**001**
Gestation at death		
28–31 weeks	–	–
32–36 weeks	1.03 (0.39; 2.94)	>0.9
37–42 weeks	**2.91 (1.34; 7.28)**	**0.012**
Mode of delivery		
Spontaneous vaginal birth	–	–
Breech	0.61 (0.03; 4.83)	0.668
Vacuum	**4.25 (1.15; 20.13)**	**0**.**039**
Caesarean section	**0.60 (0.41; 0.87)**	**0**.**008**
Location admitted from		
Another facility	–	
Community/home	1.14 (0.8; 1.61)	0.467
Condition on admission to facility		
Stable	–	
Critically ill	**0.27 (0.18; 0.39)**	**<0**.**001**
Dead on arrival	0.91 (0.46; 1.73)	0.786

Bold type denotes statistical significance.

Married women were more likely to die of PPH than an alternative cause compared with women who were single (OR 3.26, 95% CI 1.41 to 9.48). There was a trend towards increased odds of death from PPH rather than other causes with increasing parity, with the greatest risk for women being with more than three births (OR 4.62, 95% CI 1.89 to 13.9). Women who died at term (37–42 weeks of gestation) were more likely to die of PPH than an alternative cause. Vacuum delivery was significantly associated with death from PPH rather than death from an alternative cause when compared with spontaneous vaginal birth (OR 4.25, 95% CI 1.15 to 20.13) while CS was associated with reduced risk of death from PPH (OR 0.60, 95% CI 0.41 to 0.87). Women who were stable on arrival to the facility where they died were more likely to die from PPH than an alternative cause compared with those who were critically ill on arrival (OR 0.27, 95% CI 0.18 to 0.39). Age, education and the location the woman came from were not associated with odds of death from PPH compared with an alternative cause.

### Avoidable factors associated with maternal death from PPH

Maternal death case narratives were analysed to determine associated avoidable factors selected from four pre-existing categories determined by the MOH of Malawi. ‘Healthcare worker factors’ were most frequently linked with deaths from PPH (n=154 (93.4%)), followed by ‘administrative’ factors (n=95 (57.6%)), ‘patient or family’ factors (n=75, 46.1%) and ‘traditional birth attendant or community-related’ factors (n=12 (7.2%)).

**Figure 1:**
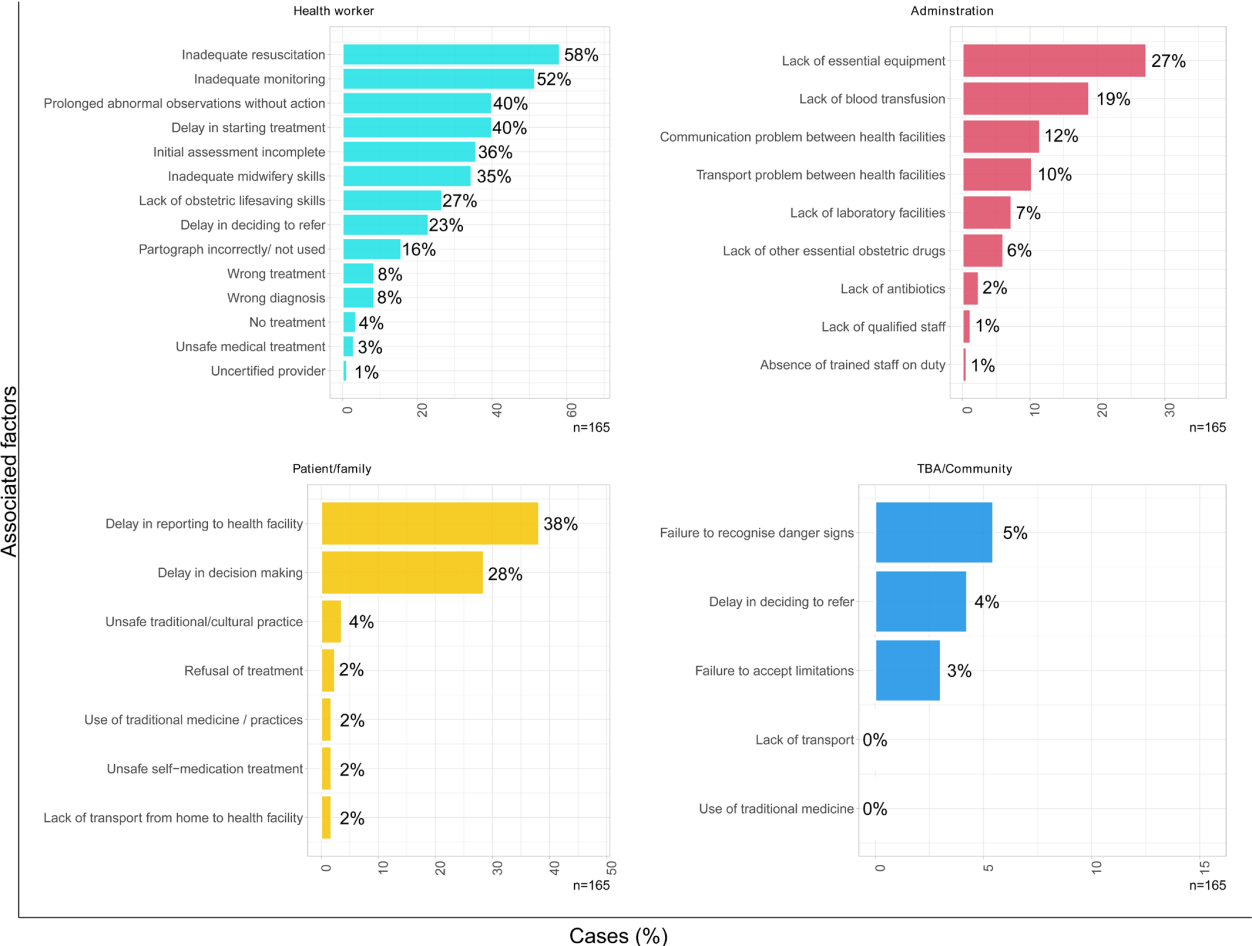
Avoidable health system factors associated with deaths from postpartum haemorrhage. TBA, traditional birth attendant.

Figure 1 shows a detailed breakdown of each category and subcategory of factors associated with PPH deaths. Factors most frequently associated with PPH deaths across all categories (figure 1) included ‘inadequate resuscitation’ (n=96 (58.2%)), ‘inadequate monitoring’ (n=85 (51.5%)), ‘prolonged abnormal observations without action’ (n=66 (40.0%)), ‘delay in starting treatment’ (n=66 (40.0%) deaths), ‘delay in patient reporting to a health facility’ (n=63 (38.2%) deaths), ‘initial assessment incomplete’ (n=59 (35.8%) deaths), ‘inadequate midwifery skills’ (n=57 (34.5%) deaths), ‘delay in decision-making’ (n=47 (28.5%) deaths), ‘lack of obstetric life-saving skills’ (n=44 (26.7%)) and ‘delay in deciding to refer’ (n=38 (23.0%) deaths).

**Figure 2:**
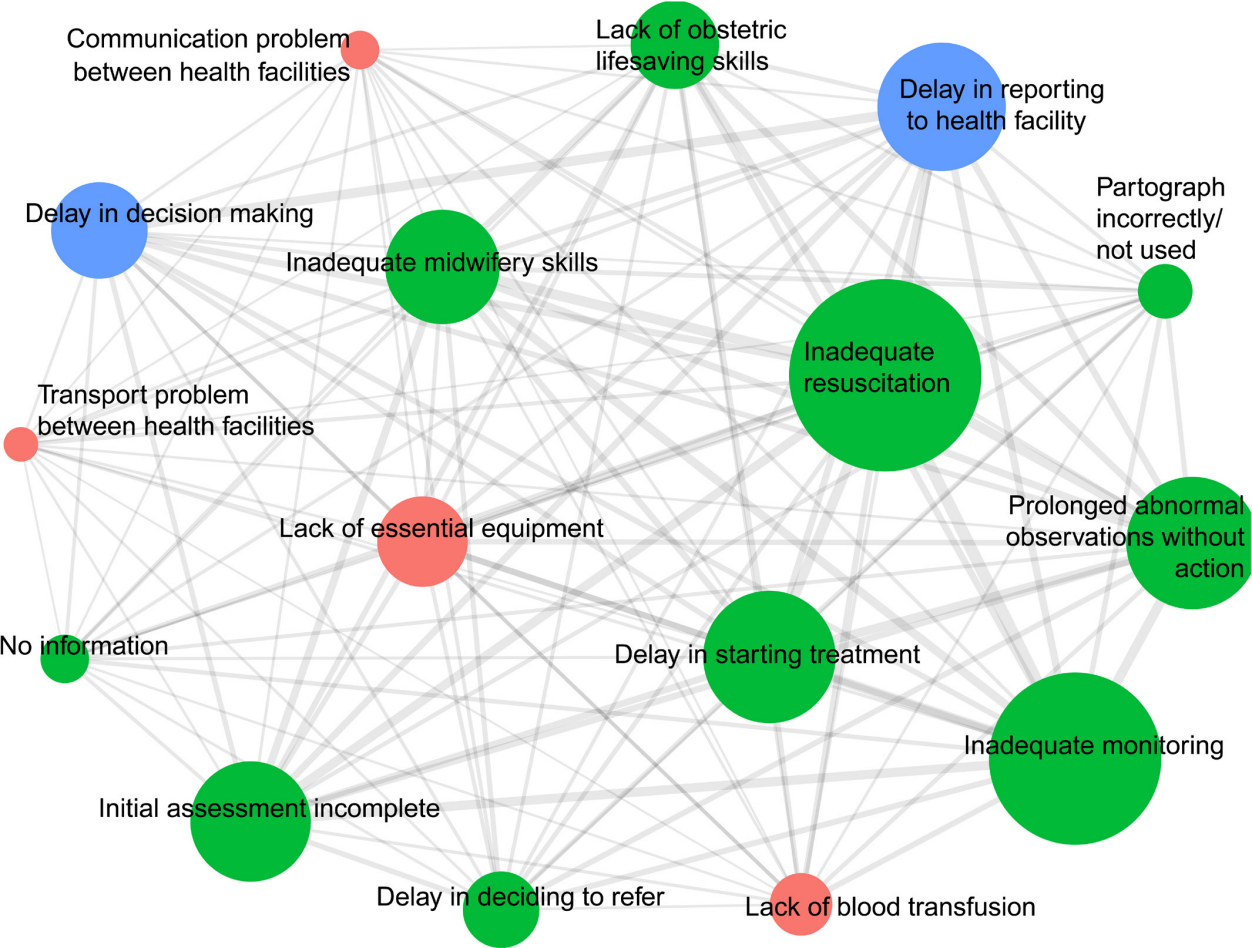
Network plot of frequently associated avoidable health system factors related to maternal deaths from postpartum haemorrhage. The size of each node shows number of cases in which each factor was presented and the thickness of lines between nodes shows the frequency of each pairing.

Figure 2 shows a network plot of avoidable factors linked to deaths from PPH with the relative size of each node representing the number of cases linked to the avoidable factor represented by the node and the weight of edges between nodes representing the number of cases with this pairing of factors. Weighting between nodes shows the frequency of association of factors in the pair. Quantitatively, the most frequent pairings were ‘inadequate monitoring’ with ‘inadequate resuscitation’ (51 of 165 cases), ‘inadequate midwifery skills’ with ‘inadequate resuscitation’ (42 cases), ‘delay in starting treatment’ with ‘inadequate resuscitation’ (41 cases) and ‘prolonged abnormal observations without action’ with ‘inadequate resuscitation’ (41 cases).

**Figure 3:**
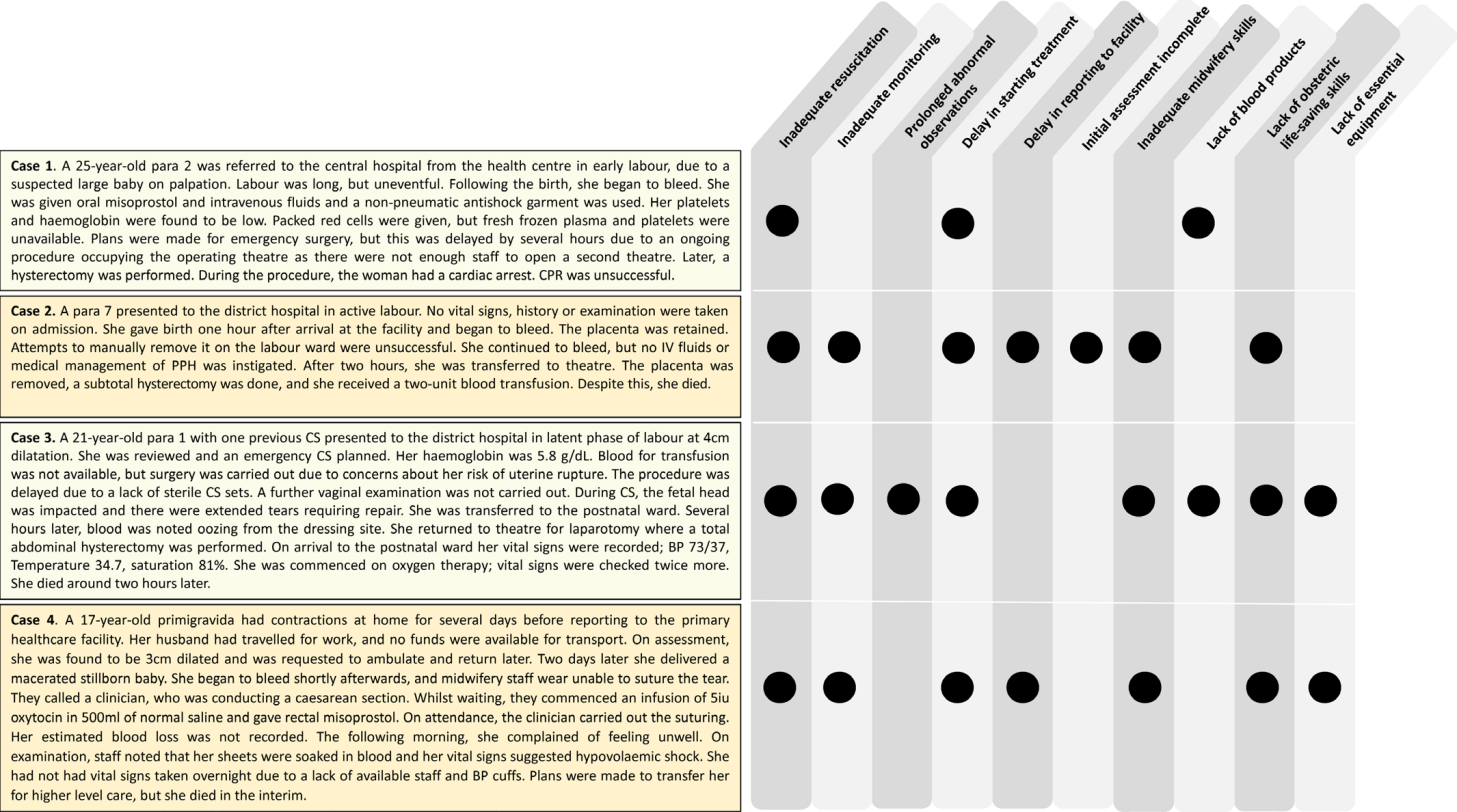
Case narratives demonstrating avoidable health system factors linked to deaths from postpartum haemorrhage in a resource-constrained setting. BP, blood pressure; CS, caesarean section; CPR, cardiopulmonary resuscitation.

Figure 3 maps case narratives to the most frequently occurring avoidable factors to add context to our findings.

### Health system factors associated with maternal death from PPH compared with death from other causes

A comparison of health system factors associated with maternal death from PPH versus those associated with deaths from other causes is contained in online supplemental table S1. Healthcare worker factors were more frequently and significantly associated with deaths from PPH compared with deaths from any other cause (154 (93.4%) of 165 vs 534 (82.9%) of 644, p<0.001). Within this category, deaths from PPH were significantly more frequently associated with several other factors compared with deaths from other causes including ‘inadequate resuscitation’ (58.2% vs 35.9%, p<0.001), ‘inadequate monitoring’ (51.5% vs 40.7%, p=0.012), ‘inadequate midwifery skills’ (34.5% vs 18.0%, p<0.001), ‘lack of obstetric lifesaving skills’ (26.7% vs 10.1%, p<0.001) and ‘partograph incorrectly or not used’ (15.8% vs 7.3%, p<0.001).

Administrative factors were also more frequently associated with deaths from PPH compared with deaths from other causes (95 (57.6%) of 165 vs 289 (44.9%) of 644, p<0.004). Deaths from PPH were significantly more frequently associated with several administrative factors compared with deaths from other causes; ‘lack of blood transfusion’ (18.8% vs 8.5%, p<0.001), ‘communication problems between facilities’ (11.5% vs 6.2%, p=0.019) and ‘transport problems between facilities’ (10.3% vs 5.3%, p=0.018).

PPH deaths were no more frequently associated with ‘patient/family-related’ factors or ‘traditional birth attendant or community-related’ factors compared with deaths from other causes.

## Discussion

We found PPH to be a leading cause of maternal death in Malawi. Avoidable ‘healthcare worker’ factors were involved in over 90% of deaths from PPH and were more strongly associated with deaths from PPH than with deaths from other causes.

Our results highlight the importance of PPH as a leading cause of maternal death in Malawi. They are in line with findings from other studies from the WHO African Region and globally determined causes of maternal death.^9 12^ In previous analyses of maternal deaths in Malawi,^13 14^ PPH was not a leading cause of death. This may be due to differences in classification systems used in reporting and lack of verification of cause of death by obstetric specialists. Our results are strengthened by the use of a robust digital data collection platform, an internationally defined classification system and a specialist review of cause of death, and therefore, provide accurate and reliable data on the causes of maternal death in Malawi, specifically highlighting the previously underestimated contribution of PPH in this setting.

Our findings indicate that women in this setting died from PPH after referral to a district hospital from another facility, after arriving in stable condition, and within the first 24 hours after giving birth; a point at which most women in this context are still inpatients. In response to global questions posed regarding bottlenecks in implementation of prevention and management strategies to reduce PPH, our data indicate that, in the Malawian context, this blockage is occurring at facility level. Malawi has successfully increased facility-based birth to over 90% coverage in recent years^15^: a notable achievement. However, reductions in maternal mortality have not mirrored the increase in facility-based skilled attendance at birth.^9^ Our data determine that deaths from PPH in hospital facilities are a problem of primary importance.

Further investigation of avoidable factors involved in deaths from PPH revealed the importance of both ‘administrative’ and ‘healthcare worker’ factors. We found that ‘administrative factors’ such as lack of infrastructure and transport and lack of resources such as drugs, blood products and essential obstetric medications were factors in most deaths from PPH and were significantly more frequently associated with maternal deaths from PPH than from other causes. This finding was expected; indeed, it is often postulated that lack of resources in healthcare facilities in resource-constrained settings is a major determinant of ongoing high rates of maternal death. Our analysis supports such factors as significant barriers to implementing strategies to prevent and manage deaths from PPH. However, we found that the association of ‘administrative’ factors with deaths from PPH was overshadowed by association with so-called ‘healthcare worker’ factors, such as ‘inadequate resuscitation’, ‘inadequate monitoring’, ‘prolonged abnormal observations without action’, ‘delay in starting treatment’ and ‘lack of obstetric life-saving skills’ in over 90% of deaths. Such avoidable factors were significantly more frequently associated with deaths from PPH than deaths from other causes, indicating that healthcare workers’ day-to-day practices and capacity may also be a significant implementation barrier. While we acknowledge that there is a crossover between lack of resources and these ‘human’ factors which must be considered when interpreting and applying our findings (eg, is monitoring inadequate because equipment is not available?), our data support the existence of a ‘know-do gap’ in the prevention and management of PPH among healthcare workers in this setting. Few previous studies examine the health system factors involved in death from PPH, though formative work undertaken prior to the recently published E-MOTIVE trial noted that ‘despite the availability of clear recommendations regarding PPH and their wide dissemination, uptake is poor at the point of care’.^16^

Surprisingly, ‘lack of qualified staff’ was not a factor found to be frequently linked to deaths from PPH. Such a finding might have been expected given the rapid increase in facility-based births in Malawi; it was recently estimated that Malawi has an unmet need of 36% in its maternity workforce.^17^ Staffing levels and qualifications to manage obstetric emergencies were not quantitatively measured in our study, and the impact of deficiencies in this area on maternal deaths from PPH warrants further investigation.

A limitation of our study design is that we could not explore the reasons for the gaps in the provision of quality care in preventing and managing PPH. However, the reasons for this are likely complicated and multifaceted, and we have attempted to outline this using case examples linked to avoidable factors. Our findings support the global prioritisation of research aimed at developing effective interventions to build capacity among front-line providers to better prevent and manage PPH. We believe there is a need for a behavioural science focus (including qualitative or ethnographic approaches) to understand more deeply and address the complexities that cause a gap between knowledge and action. As studied previously, these are common challenges in settings where resources (both material and human) are scarce and thus should be the focus of future research in such settings.^16 18^ The E-MOTIVE trial recently demonstrated the effectiveness of a bundled approach to early detection and management of PPH supported by simulation-based training, local champions, dedicated trolleys/carry cases for equipment and continuous audit and feedback in reducing PPH and deaths from PPH in resource-constrained settings.^19^ Our findings support the need for urgent roll-out of such an intervention bundle and demonstrate and quantify the avoidable factors in PPH deaths which must be tackled in the development of such approaches.

We found the risk of death from PPH compared with death from other causes reduced following CS versus vaginal birth. However, this finding is potentially misleading, as the number of women dying following CS was disproportionately high when compared with the overall CS rate for the facilities studied. The CS rate across the included facilities was 16.2%, and the overall CS rate for Malawi was last estimated at 6%,^20^ while 41.5% of women dying from PPH in our study had undergone a CS (95% CI 16.1% to 16.3%). Therefore, women undergoing CS were over-represented in deaths from PPH compared with women giving birth vaginally. Further exploration of the causes of PPH related to CS delivery in this context is required but may include prolonged labour and a high number of CS performed in the second stage of labour (see case narratives). However, we did not have data on dilatation at the time of CS, length of labour before CS nor the number of previous CS for women included in our analysis.

Vacuum delivery was associated with a higher risk of death from PPH than deaths from other causes when compared with vaginal births. This may reflect the use of vacuum delivery for a prolonged second stage of labour (itself a risk factor for PPH). However, it should also be noted that rates of assisted vaginal birth in this setting are low; unpublished data from our digital surveillance platform estimate that assisted deliveries account for only 1.2% of all births. Our findings, therefore, highlight the possibility of a need for improvement in the procedural skills and the appropriate use of vacuum birth.

We found that most women who died of PPH were admitted to the facility where they died after referral from another facility rather than from home. Given this finding, targeting interventions to better prevent and manage PPH in primary healthcare facilities may be pertinent. Our findings also indicate that most women dying of PPH in this setting are arriving at a referral centre in a stable rather than in critical condition before their deaths. This finding was unexpected and suggests that the risk of deterioration from extensive blood loss is greatest after arriving at a higher-level facility. We did not analyse the reason for referral from other facilities nor timing in relationship to birth. Although it may be assumed that women were referred after the onset of bleeding for further management of PPH, it could also be that women are referred for other reasons that may increase the risk of PPH for example, hypertension or prolonged/obstructed labour. Further analysis is planned to examine the relationship between the reason for referral, the timing of birth and death from PPH.

Deaths from PPH mainly occurred within the first 24 hours after delivery. This is a significant finding given that most women delivering in this context are still inpatients at healthcare facilities for more than 24 hours after the birth of their baby, that is, the PPH is occurring within the healthcare facility where it should be most amenable to early detection and life-saving management strategies. Findings from our study could be used to emphasise the risk of PPH, that is, highlighting to healthcare workers that women of higher parity and those undergoing CS are at particular risk of death from PPH and require closer monitoring in the postnatal period. This could be further developed into risk stratification tools prior to delivery to enable targeted prevention strategies.

Limitations of our analysis include the lack of a surviving group of women with which to compare those who died from PPH. Furthermore, we were only able to include maternal deaths which had been audited by local MDSR committees, as the standard notification form by which all deaths are reported to the MOH prior to their full audit contains insufficient information to contribute to meaningful analysis. As a result of this exclusion criteria, 30% of reported maternal deaths during the observed period were not included in the analysis presented in this paper. While 70% of all maternal deaths were audited and therefore included, there is the potential that facilities may have introduced bias in selecting which deaths to audit. From the data, we hold regarding unaudited deaths, we are unable to analyse any differences between those deaths which were audited and those which were not, other than at which facility they died. Audit rates were particularly low at two of the four central hospitals in Malawi. As such, the particular health system factors relevant to deaths from PPH at tertiary hospital level may be under-represented in our analysis. Although they represented only a small minority, data regarding the details of deaths occurring outside of the hospital setting were difficult to capture and may not be well represented in our sample as reporting and audit were more complete where deaths occurred within the facility setting.

## Conclusion

The data presented above constitute the largest published review of maternal deaths from PPH. In this setting, PPH was a leading cause of maternal death. Most women who died from this cause passed away in healthcare facilities after referral from peripheral health centres, having arrived in stable condition and within the first 24 hours of giving birth. Our findings demonstrate that key health system weaknesses are contributing to these preventable maternal deaths, particularly ‘healthcare worker’ and ‘administrative factors’, which were most frequently linked to deaths from PPH and significantly more frequently linked to PPH deaths than deaths from other causes. Our findings support the need for further investigation to identify feasible, effective interventions to reduce deaths from PPH in resource-constrained settings, with a particular focus on behavioural science and capacity building to address the ‘know-do’ gap in the context of scarce resources. Global efforts must now be focused on strategies that strengthen health systems and empower healthcare workers to reduce maternal deaths from PPH.

## Supplementary material

10.1136/bmjgh-2024-015781 online supplemental file 1

## Data Availability

Data are available on reasonable request.
