# Supplementary material for: Avoidable factors associated with maternal death from postpartum haemorrhage: a national Malawian surveillance study
Source: BMJ Glob Health. 2025 Jan 9;10(1):e015781. doi: 10.1136/bmjgh-2024-015781 (PMC11748944; doi:10.1136/bmjgh-2024-015781)
Supplement: online supplemental file 1 [file bmjgh-10-1-s001.pdf]

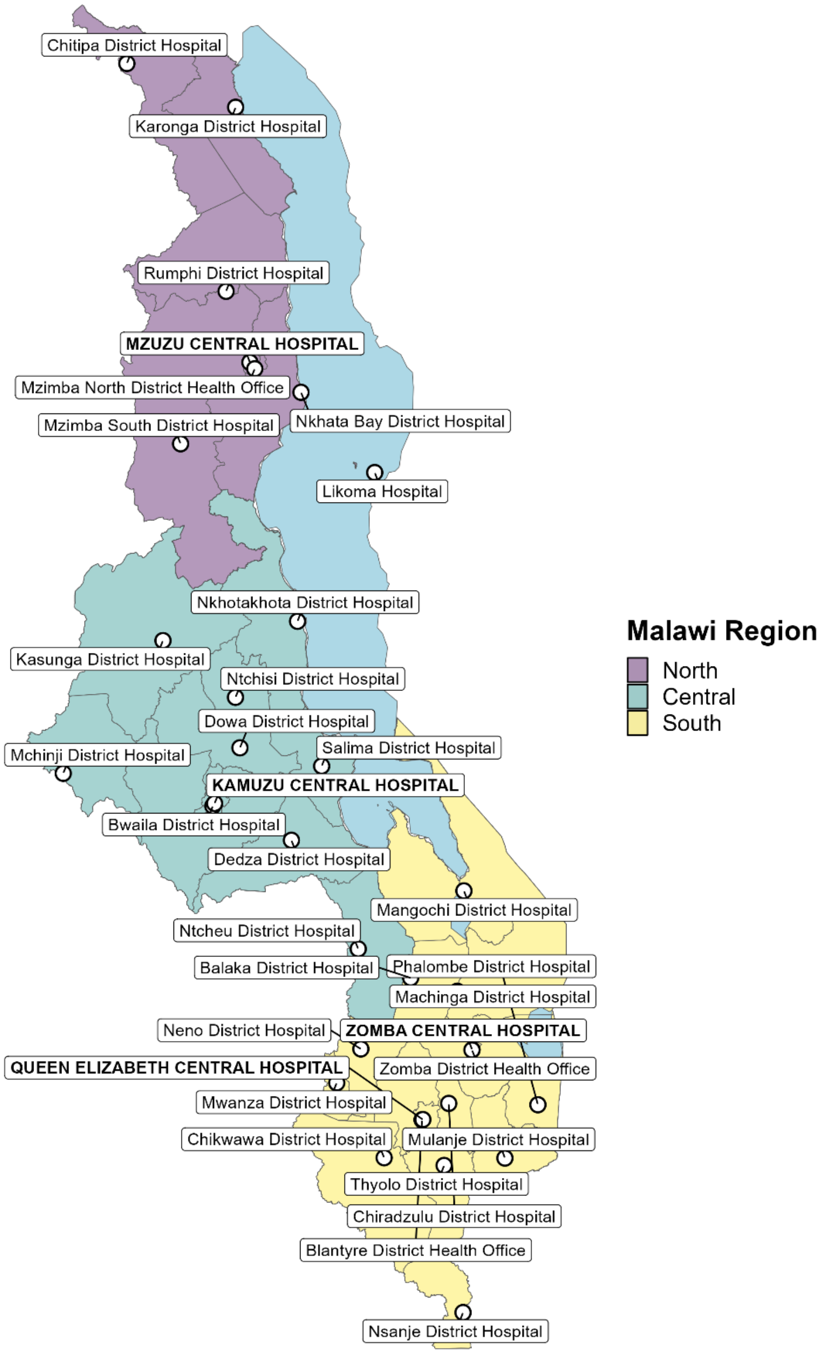

Figure S1. Map of Malawi showing facilities participating in data collection. NB Likoma Hospital is situated on an island in Lake Malawi.

	PPH<br>n=165<br>n (%)	Other causes of death<br>n=644<br>n (%)	p value
**Healthcare worker factors**			
Any healthcare worker factor	**154 (93.3%)**	**534 (82.9%)**	**<0.001**
Inadequate midwifery skills	**57 (34.5%)**	**116 (18.0%)**	**<0.001**
Uncertified provider	2 (1.2%)	3 (0.5%)	0.275
Delay in deciding to refer	38 (23.0%)	134 (20.8%)	0.534
Initial assessment incomplete	59 (35.8%)	260 (40.4%)	0.279
Inadequate resuscitation	**96 (58.2%)**	**231 (35.9%)**	**<0.001**
Wrong diagnosis	14 (8.5%)	85 (13.2%)	0.099
Partograph incorrectly/not used	**26 (15.8%)**	**47 (7.3%)**	**<0.001**
Wrong treatment	14 (8.5%)	84 (13.0%)	0.109
Unsafe medical treatment	5 (3.0%)	31 (4.8%)	0.322
No treatment	6 (3.6%)	41 (6.4%)	0.181
Delay in starting treatment	66 (40.0%)	255 (39.6%)	0.925
Inadequate monitoring	**85 (51.5%)**	**262 (40.7%)**	**0.012**
Prolonged abnormal observations without action	66 (40.0%)	210 (32.6%)	0.074
Lack of obstetric lifesaving skills	**44 (26.7%)**	**65 (10.1%)**	**<0.001**
Delay in deciding to refer	38 (23.0%)	134 (20.8%)	0.534
**Administrative factors**			
Any administrative factor	**95 (57.6%)**	**289 (44.9%)**	**0.004**
Communication problems between facilities	**19 (11.5%)**	**40 (6.2%)**	**0.019**
Transport problems between facilities	**17 (10.3%)**	**34 (5.3%)**	**0.018**
Lack of qualified staff	2 (1.2%)	19 (3.0%)	0.210
Lack of antibiotics	4 (2.4%)	11 (1.7%)	0.543
Lack of essential obstetric drugs	10 (6.1%)	34 (5.3%)	0.693
Lack of essential equipment	45 (27.3%)	137 (21.3%)	0.100
Lack of laboratory facilities	12 (7.3%)	54 (8.4%)	0.641
Lack of blood transfusion	**31 (18.8%)**	**55 (8.5%)**	**<0.001**
Absence of trained staff on duty	1 (0.6%)	17 (2.6%)	0.114
**Patient or Family factors**			
Any patient or family associated factors	75 (45.5%)	315 (48.9%)	0.428

Delay in reporting to the health facility	63 (38.2%)	243 (37.7%)	0.916
Lack of transport from home to facility	3 (1.8%)	15 (2.3%)	0.691
Unsafe traditional/cultural practices	6 (3.6%)	22 (3.4%)	0.890
Unsafe self-medication	3 (1.8%)	22 (3.4%)	0.290
Refusal of treatment	4 (2.4%)	32 (5.0%)	0.157
Delay in decision-making	47 (28.5%)	183 (28.4%)	0.986
Use of traditional medicine/practices	3 (1.8%)	20 (3.1%)	0.375
**TBA/Community factors**			
Any traditional birth attendant or Community factor	12 (7.3%)	60 (9.3%)	0.411
Failure to recognise danger signs	9 (5.5%)	42 (6.5%)	0.615
Failure to accept limitations	5 (3.0%)	18 (2.8%)	0.871
Use of traditional medicine	0 (0.0%)	7 (1.1%)	0.179
Lack of transport	0 (0.0%)	3 (0.5%)	0.380
Delay in deciding to refer	7 (4.2%)	22 (3.4%)	0.610

Table S1. Association of health system factors with deaths from PPH *versus* deaths from other causes
